# Antifungal Activity of Coumarin Against *Candida albicans* Is Related to Apoptosis

**DOI:** 10.3389/fcimb.2018.00445

**Published:** 2019-01-04

**Authors:** Chang Jia, Jian Zhang, Lili Yu, Chenglu Wang, Yijia Yang, Xing Rong, Ke Xu, Maoping Chu

**Affiliations:** ^1^Pediatric Research Institute, The Second Affiliated Hospital and Yuying Children's Hospital of Wenzhou Medical University, Wenzhou, China; ^2^Children's Heart Center, Institute of Cardiovascular Development and Translational Medicine, The Second Affiliated Hospital and Yuying Children's Hospital of Wenzhou Medical University, Wenzhou, China; ^3^The Second Clinical Medical College of Wenzhou Medical University, Wenzhou, China; ^4^The Institute of Life Sciences, Wenzhou University, Wenzhou, China

**Keywords:** coumarin, *Candida albicans*, apoptosis, ROS, Ca^2+^

## Abstract

Coumarin (1,2-benzopyrone), an aromatic oxygen-containing heterocyclic compound, has various biological functions. Previous studies have demonstrated that coumarin and its derivatives exhibit antifungal activity against *Candida albicans*. In this study, we investigated the exact mechanism by which coumarin works against this fungus using Annexin V-FITC/PI double staining, TUNEL assay, and DAPI staining, and found that it induced a series of apoptotic features, including phosphatidylserine (PS) externalization, DNA fragmentation, and nuclear condensation. Moreover, it also induced cytochrome *c* release from the mitochondria to the cytoplasm and metacaspase activation. Further study revealed that intracellular reactive oxygen species (ROS) levels were increased and mitochondrial functions, such as mitochondrial membrane potential and mitochondrial morphology, were altered after treatment with coumarin. Cytosolic and mitochondrial Ca^2+^ levels were also found to be elevated. However, pretreatment with ruthenium red (RR), a known mitochondrial Ca^2+^ channel inhibitor, attenuated coumarin-mediated DNA fragmentation and metacaspase activity, indicating that the coumarin-induced *C. albicans* apoptosis is associated with mitochondrial Ca^2+^ influx. Finally, coumarin was found to be low-toxic and effective in prolonging the survival of *C. albicans*-infected mice. This study highlights the antifungal activity and mechanism of coumarin against *C. albicans* and provides a potential treatment strategy for *C. albicans* infection.

## Introduction

*Candida albicans* is a commensal fungus that normally inhabits the human gastrointestinal tract and skin. However, in individuals who are immunocompromised due to AIDS or cancer chemotherapy, *C. albicans* can cause severe mucosal infections as well as fatal invasive infections (Ganguly and Mitchell, 2011). In the USA, fungal bloodstream infections caused by *Candida* spp. rank fourth among the nosocomial infections with considerable mortality rates (Edmond et al., 1999; Wisplinghoff et al., 2004). The presently used antifungal drugs mainly include azoles, polyenes, allylamines, echinocandins, and 5-fluorocytosine. With the large-scale application of broad-spectrum antifungal agents, the prevalence of opportunistic pathogen infections has gradually increased. Therefore, exploring novel and more effective antifungals is required to cope with lethal candidiasis.

Natural products, particularly traditional Chinese medicine, provide a rich pool for drug discovery due to the therapeutic efficacy and versatile structures of their secondary metabolites. Coumarin (1,2-benzopyrone) is an aromatic oxygen-containing heterocyclic compound containing benzopane α-pyranone structures that can be isolated from Solanaceae, Rutaceae, Umbelliferae, and other plants, or totally synthesized in the laboratory. Previous studies have demonstrated that coumarin and its derivatives have a variety of biological activities, including anticoagulant, antibacterial, and antiviral activities (Jung and Park, 2009). Additionally, coumarin complexes inhibit soil-borne fungal pathogens, plant fungal pathogens, and human fungal pathogens such as *C. albicans, C. tropicalis*, and *Aspergillus fumigatus* (Kumar et al., 2012; Hu et al., 2017). Although exposure to coumarin for 24 h reportedly alters the cell membrane and cell wall, and decreases the cytoplasmic volume with structural disorganization in *C. albicans*, the exact mechanism of action of coumarin (1,2-benzopyrone) has yet to be delineated (Widodo et al., 2012). Previous studies have reported that the silver (I)-coumarin complexes exert fungicidal activity against *C. albicans* by disrupting cytochrome synthesis, which then leads to respiration inhibition and reduced ergosterol biosynthesis. Moreover, a loss of cytochrome *c* synthesis (or depletion of its levels within the cells) also results in apoptosis. However, the inhibition of cytochrome synthesis by silver (I)-coumarin complexes is mainly attributed to silver addition (Thati et al., 2007). Previous studies have also demonstrated that coumarin or its derivatives exert anti-cancer activity, which is associated with apoptosis (Chuang et al., 2007; Álvarez-Delgado et al., 2009; Musa et al., 2012). However, it is still unknown whether this type of coumarin (i.e., 1,2-benzopyrone) exerts its antifungal activity against *C. albicans* via apoptosis.

Apoptosis is a form of programmed cell death. In addition to multicellular organisms, unicellular organisms, such as yeast, are also able to undergo programmed cell death that exhibits many hallmarks of apoptosis, including phosphatidylserine (PS) externalization, DNA fragmentation, metacaspase activation, reactive oxygen species (ROS) accumulation, loss of mitochondrial membrane potential, and elevations in cytosolic and mitochondrial Ca^2+^ (Leadsham et al., 2010). In this study, a variety of the aforementioned apoptosis markers were measured in *C. albicans* cells after treatment with this compound to investigate the antifungal mechanism of coumarin (1,2-benzopyrone) against *C. albicans*.

## Materials and Methods

### Chemicals

Coumarin (1,2-benzopyrone, >99%; CAS-No. 91-64-5) used in this study was purchased from Sangon Biotech. (Shanghai, China). A stock solution of coumarin (100 mg/mL) was prepared in dimethyl sulfoxide (DMSO) and then diluted in culture medium to obtain the concentrations to be tested (keeping the final concentration of DMSO at no more than 2%).

### Microorganism and Media

*C. albicans* SC5314 (ATCC MYA-2876) was obtained from the American Type Culture Collection (ATCC). The fungal strain cultures were routinely maintained in YPD medium (1% yeast extract, 2% peptone and 2% dextrose) at 30°C.

### Antifungal Susceptibility Testing

*C. albicans* suspensions (1 × 10^6^ cells/mL) containing various concentrations of coumarin were dispensed into 96-well microtiter plates at a volume of 0.1 mL/well. After 48 h of incubation at 30°C, the minimal inhibitory concentration (MIC), which is the lowest concentration of the compound that prevents visible growth of fungus, was determined (Tian et al., 2017a,b; Yun and Lee, 2017). Each test was performed in triplicate.

*C. albicans* cells (1 × 10^7^ cells/mL) containing three concentrations of coumarin (0.5, 1.0, and 2.0 mg/mL) were dispensed into 96-well microtiter plates at a volume of 0.1 mL/well. After 24 h and 48 h of incubation at 30°C, the optical density at 600 nm (OD_600_) was measured.

After incubation with coumarin (0.5, 1.0, and 2.0 mg/mL) for 4 h, the cultures were acquired and spread onto YPD agar plates. Colony-forming units (CFUs) were counted after incubation for 24 h at 30°C. The percentage survival was determined relative to the untreated cells. All experiments were performed three times independently.

### Analysis of Phosphatidylserine (PS) Externalization, DNA Fragmentation, and Nuclear Condensation

Overnight cultures were refreshed in YPD medium to mid-exponential phase, then treated with 0.5, 1.0, and 2.0 mg/mL coumarin for 4 h. After that, the cells were harvested. To prepare protoplasts, *C. albicans* cells were incubated at 35°C for 20 min in 0.02 mg/mL Zymolyase 20T in 0.1 M potassium phosphate buffer (PPB, 50 mM K_2_HPO_4_, 5 mM EDTA, 50 mM DTT, 50 mM KH_2_PO_4_, and 40 mM β-mercaptoethanol) with 2.4 M sorbitol and at pH7.2. Thereafter, 100 μL permeabilization solution (0.1 M sodium citrate (pH6.0) with 0.1% Triton X-100) was added to the washed protoplasts, which were then plated on ice for 2 min and washed again. Next, the AnnexinV-FITC apoptosis detection kit was used according to the manufacturer's instructions. The stained cells were analyzed by flow cytometry.

DNA fragmentation and condensation were examined using TUNEL and DAPI staining (Park and Lee, 2010). The treated cells were washed twice with phosphate-buffered saline (PBS), then fixed in 3.6% paraformaldehyde for 30 min, and permeabilized on ice for 2 min. After the cells were washed again with PBS, they were stained using an *in situ* cell death detection kit for 1 h at 37°C and assessed by flow cytometer. Nuclear fragmentation and condensation were examined with DAPI staining. After incubation with coumarin for 4 h, the cells were harvested, washed and resuspended in PBS, stained with 1 μg/mL DAPI in the dark for 20 min, then washed three times with PBS and examined by fluorescence microscopy.

### Assessment of Cytochrome *c* Release

Cytochrome *c* levels were examined as described previously with minor modification (Choi and Lee, 2015; Yun and Lee, 2016). The treated *C. albicans* was harvested and prepared for protoplasts according to the abovementioned procedures. After that, the protoplasts were homogenized in buffer A (50 mM Tris, 2 mM EDTA, 1 mM phenylmethylsulfonyl fluoride, pH7.5), and the supernatant was collected. To analyze cytosolic and mitochondrial cytochrome *c* levels, the obtained supernatant was centrifuged at 50,000 rpm for 45 min. The released cytoplasmic cytochrome *c* was measured in the supernatant. To get pure mitochondria, the pellet was suspended in buffer B (50 mM Tris, 2 mM EDTA, pH 5.0). After treatment with 500 mg/mL ascorbic acid for 5 min, the cytochrome *c* content in the cytoplasmic or mitochondrial samples was determined by spectrophotometric analysis at 550 nm. The protein content was quantified by the Bradford method.

### Metacaspase Activation Assay

Metacaspase activation was examined using the CaspACE FITC-VAD-FMK *in situ* marker (Promega) (Tian et al., 2017b). The treated *C. albicans* cells were centrifuged, washed and then stained with 10 μM CaspACE FITC-VAD-FMK at 30°C for 30 min in the dark. After that, the cells were washed again and examined using flow cytometry.

### ROS Measurement

To detect the production and accumulation of ROS, *C. albicans* cells were analyzed using 2′, 7′- dichlorofluorescein diacetate (DCFH-DA) (Jia et al., 2014). Cells exposed to coumarin for 4 h were harvested by centrifugation, washed once with YPD, and then resuspended in 0.5 mL YPD. DCFH-DA was added at a final concentration of 10 μM and incubated at 30°C for 30 min in the dark. The fluorescence intensities of resuspended cells were determined via flow cytometry.

### Analysis of Mitochondrial Membrane Potential

5,5′,6,6′-Tetrachloro-1,1′,3,3′-tetraethyl-benzimidazolyl carbocyanine iodide (JC-1; Molecular Probes) was used to examine changes in mitochondrial membrane potential (Choi and Lee, 2015). *C. albicans* cells treated with coumarin for 4 h were washed, and stained with 2.5 μg/mL JC-1 for 20 min in the dark. After washed in PBS, the cells were analyzed by flow cytometry. The ratio of the fluorescence intensities of aggregates JC-1 (FL2) to monomer (FL1) was calculated.

### Cytosolic and Mitochondrial Calcium Determination

The effects of coumarin on cytosolic and mitochondrial calcium were tested using Fluo-3 AM and Rhod-2 AM, respectively (Tian et al., 2017b). Briefly, the treated *C. albicans* cells were harvested and washed twice with HBSS buffer, and then resuspended in 500 μL HBSS buffer. For cytosolic calcium measurement, Fluo-3 AM (prepared in 0.1% Pluroinc F-127 solution in DMSO) was added to a final concentration of 2 μM. After incubation at 30°C for 40 min in the dark, the cells were washed once, resuspended in 600 μL HBSS and incubated at 30°C for further 20 min. For mitochondrial calcium measurement, Rhod-2 AM (prepared in 0.05% Pluroinc F-127 solution in DMSO) was added to a final concentration of 4 μM, and incubated at 37°C for 30 min in darkness. The fluorescence densities of Fluo-3 AM (excitation = 480 nm, emission = 526 nm), and Rhod-2 AM (excitation = 550 nm, emission = 580 nm) were immediately measured using flow cytometry.

### Murine Model of *C. albicans* Infection

To demonstrate whether coumarin was capable of clearing *C. albicans* infection, C57BL/6 mice were infected via the tail vein with 100 μL of a 5 × 10^6^ *C. albicans* cells/mL normal saline suspension, and then randomly placed into four groups: normal saline group, DMSO group, 20 mg/kg coumarin group, and 40 mg/kg coumarin group. After 1 h infection, 100 μL of normal saline, DMSO, 20 mg/kg coumarin and 40 mg/kg coumarin were, respectively, administered by oral-gastric (OG) gavage, and then repeated on day 2 and day 3. Six mice were used for each group. The survival of mice was evaluated on day 10. To evaluate the toxicity of coumarin, male C57BL/6 mice aged 6–8 weeks were administrated by OG gavage with coumarin at doses of 20 and 40 mg/kg body weight once a day for 3 days. Each group included six mice. Survival was monitored for 10 days. The Kaplan–Meier log-rank test was used to determine the statistical differences between groups.

### Statistical Analysis

Every experiment was independently conducted at least three times, and data were presented as mean ± SD. The results were analyzed by SPSS software, using Duncan's Multiple Range test following one-way ANOVA. The statistically significant *P* values were noted with asterisks (^*^ *P* < 0.05, ^**^ *P* < 0.01, and ^***^ *P* < 0.001).

## Results

### Effects of Coumarin on Cell Growth and Survival in *C. albicans*

**Figure 1:**
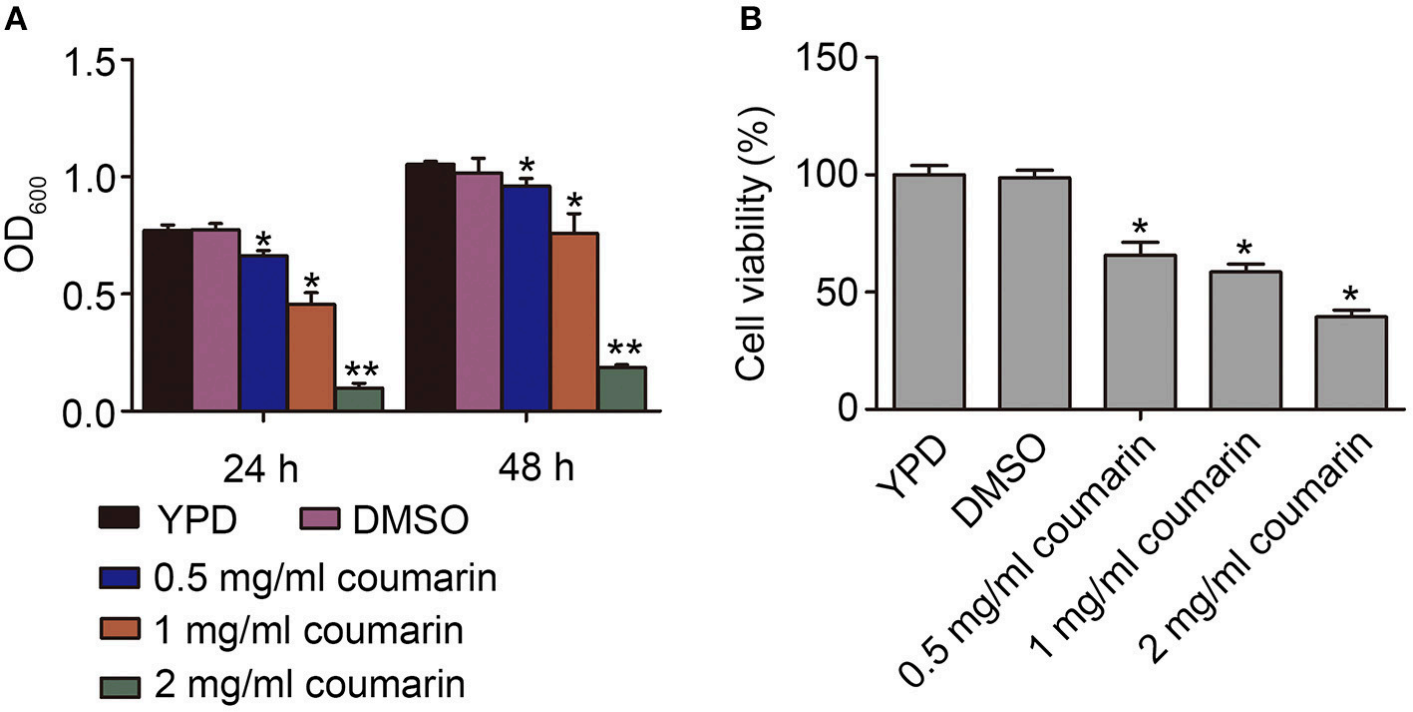
Effects of coumarin on growth and viability of *Candida albicans*. **(A)** The OD_600_ value was measured after treatment with coumarin for 24 h and 48 h. **(B)** Cell viability was assessed by counting CFUs after treatment with different concentrations of coumarin for 4 h at 30°C. Data were shown as mean ± SD. *P < 0.05 and **P < 0.01.

The antifungal activity of coumarin against *C. albicans* was studied (Figure S2). The MIC for the SC5314 strain was 2.0 mg/mL. To determine the effect of coumarin on strain growth, OD_600_ of the strain was measured after treatment with 0.5, 1.0, and 2.0 mg/mL coumarin for 24 and 48 h. The results showed that the growth of the strains was significantly inhibited by coumarin in a dose-dependent manner (Figure 1A). To evaluate whether the decreased growth was related to cell death, the survival of the strains was examined after pretreatment with coumarin for 4 h. The CFUs assays showed that the survival rate was remarkably lowered by coumarin (Figure 1B). Taken together, these results indicated that the growth inhibition by coumarin was associated with reduced cell survival.

### Coumarin Triggers PS Externalization, DNA Fragmentation, and Nuclear Condensation

**Figure 2:**
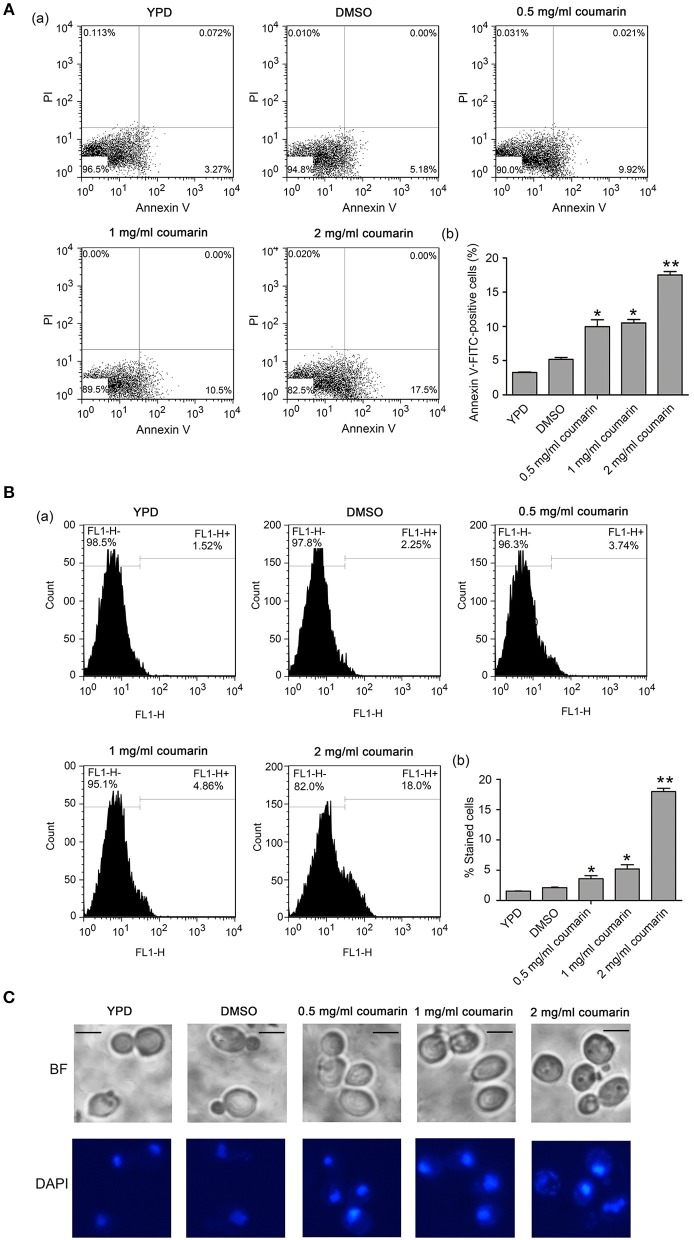
Apoptosis was induced by coumarin treatment for 4 h. **(A)** Phosphatidylserine (PS) externalization was evaluated by Annexin V-FITC and PI double staining. The histogram was the quantitative analysis of Annexin-FITC-positive cells, and the data were shown as mean ± SD. *P < 0.05 and **P < 0.01. **(B)** DNA fragmentation was observed by TUNEL assay using flow cytometry. The histogram displayed the percentage of stained cells, and the values were expressed as mean ± SD, *P < 0.05 and **P < 0.01. **(C)** Nuclear condensation was observed by DAPI staining. BF, Bright Field. Bar = 5 μm.

The inhibition of yeast proliferation and cell survival might result from induced yeast apoptosis (Almeida et al., 2008). In normal cells, phosphatidylserine (PS) exists in the inner leaflet of the plasma membrane. However, PS is exposed on the outer leaflet in apoptotic and necrotic cells, which is assumed to be an early marker of apoptosis in fungi (Choi and Lee, 2015; Tian et al., 2017a). Here, the *C. albicans* cells treated with coumarin were observed using AnnexinV-FITC and PI co-staining (Vermes et al., 2000). AnnexinV specifically binds to externalized PS, and PI can be used to examine cell membrane integrity. Apoptotic cells are Annexin V-FITC+/PI-, whereas necrotic cells are V-FITC+/PI+ (Choi and Lee, 2015). In this study, after treatment with different concentrations of coumarin, the number of cells in the Annexin V+/PI- quadrants increased, whereas the number of cells in the Annexin V+/PI+ quadrants remained almost unchanged (Figure 2A), revealing that coumarin induced *C. albicans* apoptosis.

As another apoptosis hallmark, DNA fragmentation can be mediated by the pro-apoptotic factors, Aif1 and Nuc1 (Madeo et al., 2009). To investigate whether coumarin could lead to DNA fragmentation, the treated cells were assessed using TUNEL assay, which could detect apoptotic DNA cleavage by labeling fluorescent dUTP at the 3'-OH ends of DNA (Phillips et al., 2003). An enhanced fluorescence intensity was observed with an increase in coumarin concentration (Figure 2B), suggesting that coumarin caused DNA fragmentation. To monitor the morphological changes in the nucleus, a DAPI assay was conducted. Fluorescence microscopy analysis revealed that cells treated with coumarin showed a more concentrated or cleaved fluorescence when compared to the control cells (Figure 2C), implying nuclear condensation and fragmentation.

Altogether, these results indicated that coumarin treatment led to PS externalization, DNA fragmentation, and nuclear condensation, which are key features of apoptosis.

### Coumarin Induces Cytochrome *c* Release and Metacaspase Activation

**Figure 3:**
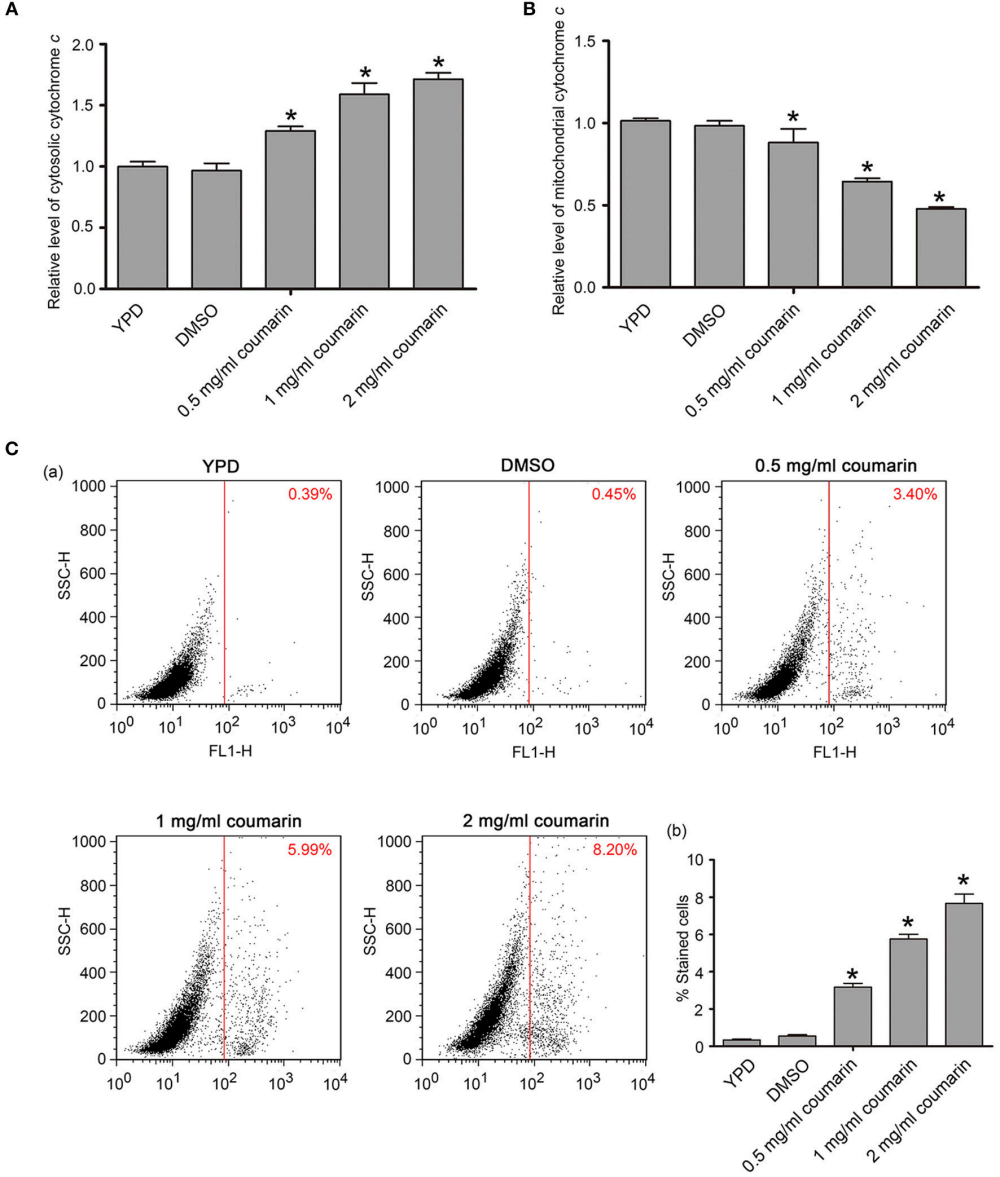
Cytochrome *c* release and metacaspase activation were assayed after coumarin treatment for 4 h. **(A,B)** Cytosolic **(A)** and mitochondrial **(B)** cytochrome *c* levels were, respectively, measured. **(C)** Metacaspase activity was assessed by FITC-VAD-FMK assay. The percentage of stained cells was shown in the histogram, and the data were presented as mean ± SD. *P < 0.05.

Cytochrome *c* release from the mitochondria to the cytoplasm is a crucial event, which activates metacaspase, thereby inducing apoptosis (Wu et al., 2010). Here, the translocation of cytochrome *c* from the mitochondria and metacaspase activity were examined. Compared to the control cells, the relative levels of cytosolic cytochrome *c* were observed to have increased while mitochondrial cytochrome *c* contents were simultaneously observed to have decreased upon coumarin treatment (Figures 3A,B), thereby revealing that coumarin triggered the release of cytochrome *c* from the mitochondria to the cytosol.

Metacaspases, which are caspase-like cysteine proteases in yeast, play a central role in the early stages of apoptosis and can be detected via FITC-VAD-FMK staining (Park and Lee, 2010). Cells with activated intracellular metacaspases exhibit fluorescence, while the control cells appear unstained. Fluorescence intensity enhanced as the concentration of coumarin increased (Figure 3C), suggesting that metacaspase activity was elevated in coumarin-treated cells.

### Coumarin Increases ROS Levels and Affects Mitochondrial Membrane Potential and Morphology in *C. albicans*

**Figure 4:**
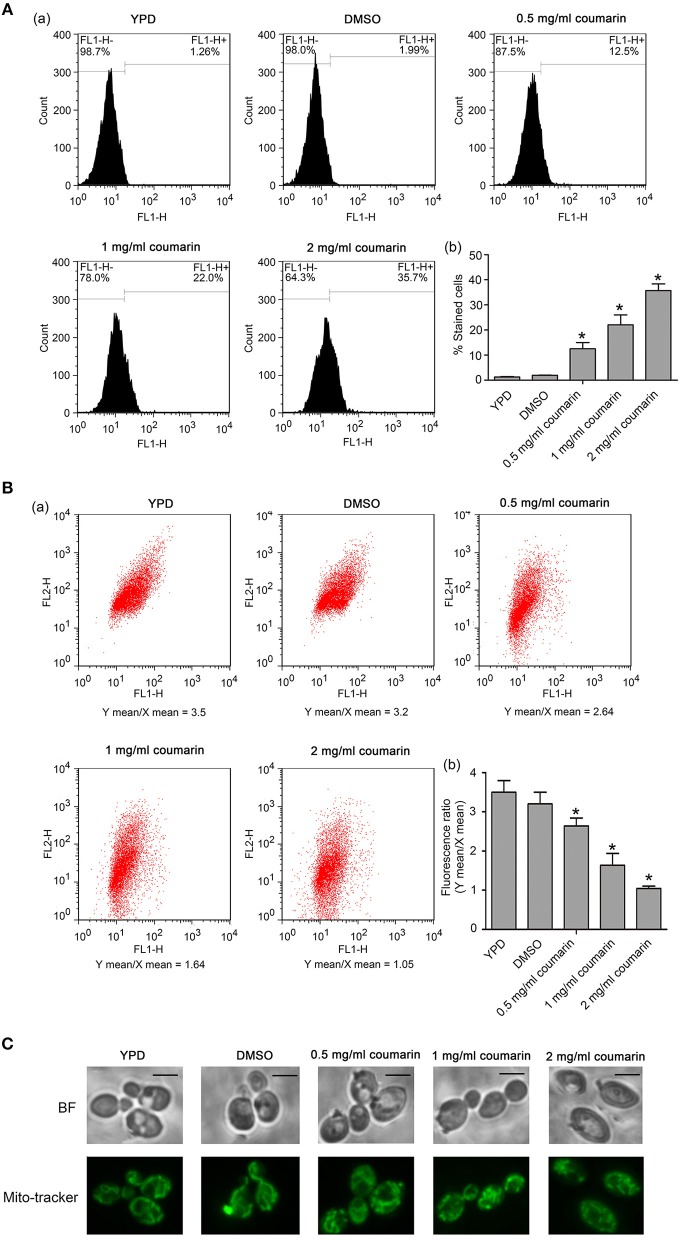
ROS levels and mitochondrial functions were detected in *C. albicans*. **(A)** The intracellular ROS levels were analyzed using DCFH-DA. The histogram represented the percentage of stained cells, and the data were shown as mean ± SD, *P < 0.05. **(B)** Mitochondrial membrane potential was evaluated using JC-1 staining. The histogram was the quantitative result of fluorescence ratio (Y mean/X mean). *P < 0.05. **(C)** Mitochondrial morphology was observed using Mito-Tracker Green. BF, Bright Field. Bar, 5 μm.

ROS play an essential role in yeast apoptosis since they induce and regulate apoptosis (Madeo et al., 1999). Thus, we used the fluorescent probe DCFH-DA, a ROS indicator, to assess intracellular ROS levels. Compared to the control cells, coumarin notably increased the fluorescence intensity in a dose-dependent manner (Figure 4A), suggesting that coumarin caused ROS accumulation in *C. albicans*. Elevated ROS levels can oxidize unsaturated fatty acids and trigger lipid peroxidation to form products such as malondialdehyde (MDA) (Mann et al., 2018). In our data, MDA was remarkably increased upon coumarin treatment (Figure S1), further confirming ROS accumulation in coumarin-treated cells.

Mitochondria also play an important role in yeast apoptosis since this organelle is the major site for ROS production and contains many proapoptotic factors such as cytochrome *c*, Aif1p, and Nuc1p (Pereira et al., 2008). The loss of mitochondrial membrane potential and enhancement of mitochondrial permeability are hallmarks of the early stages of apoptosis (Choi and Lee, 2015), and we assayed mitochondrial involvement in coumarin-induced apoptosis via JC-1 staining. In the mitochondrial matrix of normal cells, the lipophilic cationic JC-1 dye is observed as red aggregates. Contrastingly, in apoptotic cells with depolarized mitochondria, JC-1 remains in the cytoplasm and appears green. As shown in Figure 4B, FL2/FL1 ratio decreased in coumarin-treated cells in a concentration-dependent manner, suggesting that coumarin caused mitochondrial depolarization in *C. albicans*. Furthermore, mitochondrial morphology observation found that coumarin caused morphologically abnormal mitochondria that mostly had a fragmented appearance (Figure 4C). Taken together, coumarin addition affected mitochondrial functions.

### Coumarin Elevates Cytosolic and Mitochondrial Ca^2+^ Levels

**Figure 5:**
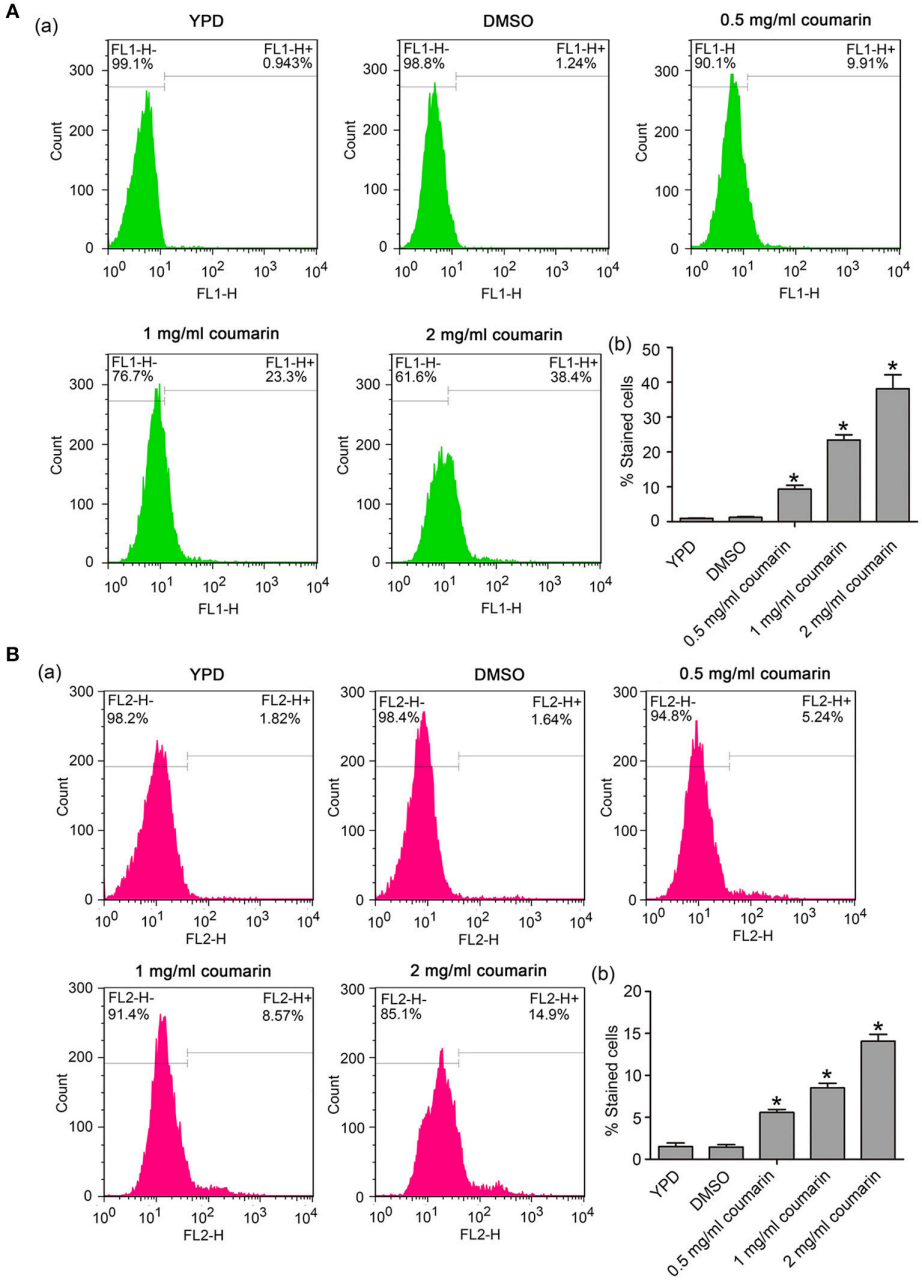
Ca^2+^ levels were, respectively, examined in the cytosol and mitochondria. **(A)** Cytosolic Ca^2+^ contents were determined using Fluo-3 AM staining. The histogram showed the percentage of stained cells, and the data were shown as mean ± SD. *P < 0.05. **(B)** Mitochondrial Ca^2+^ levels were evaluated via Rhod-2 AM staining. The histogram was the quantitative analysis of the percentage of stained cells, and the data were presented as mean ± SD. *P < 0.05.

Besides ROS, Ca^2+^ also plays an important role in initiating and executing yeast apoptosis (Carraro and Bernardi, 2016). Increased cytosolic Ca^2+^ triggers mitochondrial permeabilization and releases proapoptotic factors, which initiate the programmed cell death. In this study, Fluo-3 AM and Rhod-2 AM were used to measure cytosolic and mitochondrial Ca^2+^ levels, respectively. Data showed that the fluorescence intensities of both Fluo-3 AM and Rho-2 AM were observed to significantly increase in *C. albicans* cells treated with coumarin (Figures 5A,B), suggesting that coumarin triggered a movement of Ca^2+^ and its accumulation in the cytosol and mitochondria.

### Coumarin Induces Apoptosis Related to Mitochondrial Ca^2+^ Influx

**Figure 6:**
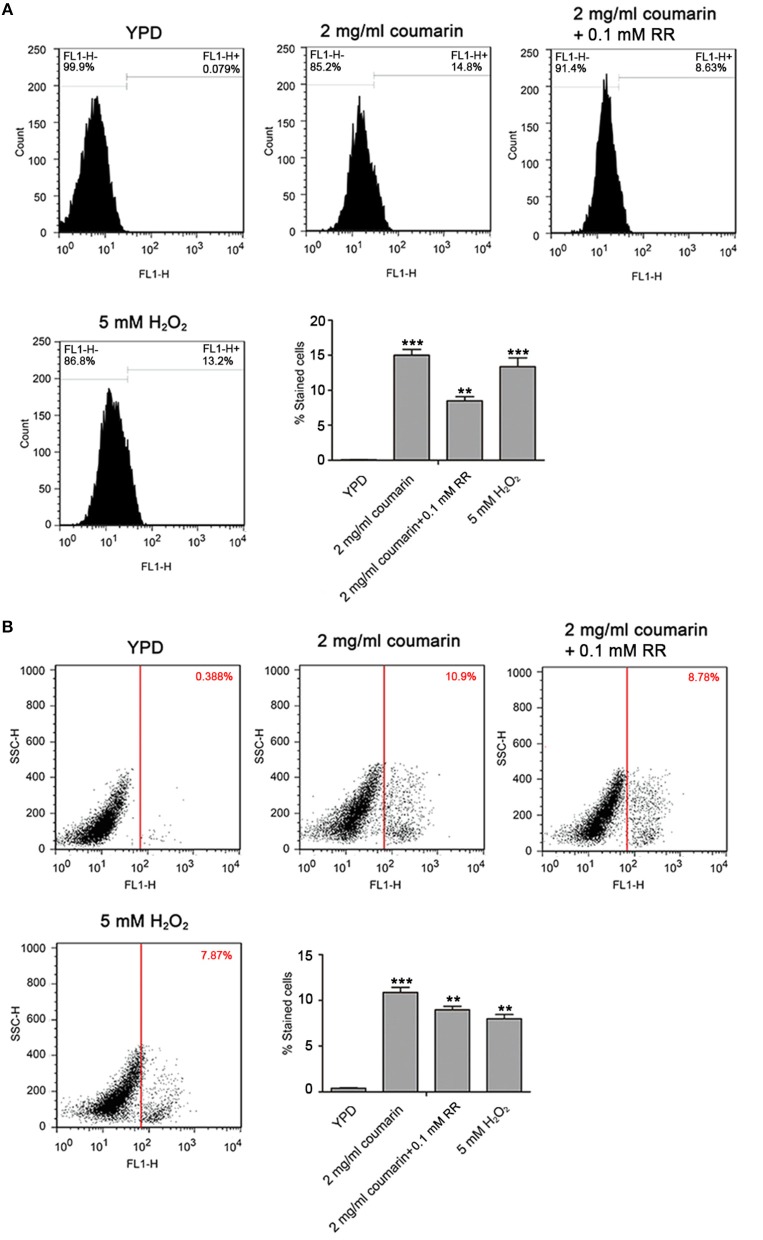
Coumarin triggered apoptosis associated with mitochondrial Ca^2+^ influx. **(A)** DNA fragmentation was analyzed using TUNEL staining. The histogram displayed the percentage of stained cells, and the values were exhibited as mean ± SD, **P < 0.01 and ***P < 0.001. **(B)** Metacaspase activity was evaluated using FITC-VAD-FMK assay. The percentage of stained cells was shown in the histogram, and the data were displayed as mean ± SD. **P < 0.01 and ***P < 0.001.

An increase in ROS and Ca^2+^ is both an event of apoptosis, and plays a crucial role in the process of apoptosis (Zhang et al., 2008; Yun and Lee, 2016; Lee and Lee, 2017). To determine whether apoptosis caused by coumarin was related to increased ROS or mitochondrial Ca^2+^, a pretreatment with 5 mM *N*-acetyl cysteine (NAC), a ROS scavenger, and 0.1 mM ruthenium red (RR) (Lupetti et al., 2004; Yun and Lee, 2016), a mitochondrial Ca^2+^ influx inhibitor, was conducted to determine DNA fragmentation and metacaspase activity. Results showed that ROS elimination did not rescue apoptosis (data not shown). However, inhibition of the mitochondrial Ca^2+^ influx remarkably decreased DNA fragmentation and metacaspase activity (Figures 6A,B and Figures S3, S4). These results demonstrated that coumarin-induced *C. albicans* apoptosis was relevant to elevated mitochondrial Ca^2+^ levels.

### Coumarin Rescues the Murine Model of *C. albicans* Infection

**Figure 7:**
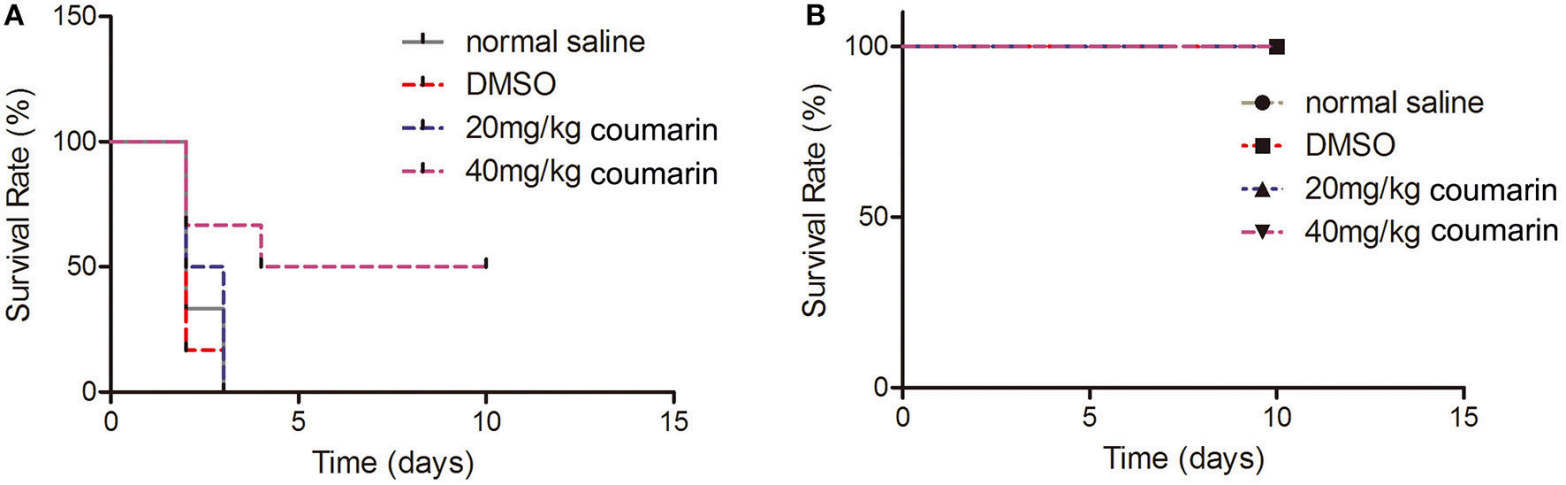
The effects of coumarin on the survival of mice were evaluated. **(A)** Survival rate of *C. albicans*-infected mice was assessed after exposure to coumarin for 10 days. **(B)** Toxicity of coumarin was observed in non-infected mice.

To examine the effects of coumarin on the virulence of *C. albicans in vivo*, we administrated coumarin by oral-gastric (OG) gavage in a mouse model of systemic candidiasis. The results showed that mice in the normal saline, DMSO, and 20 mg/kg coumarin groups died within 3 days, while 50% of mice treated with 40 mg/kg coumarin survived the complete course of the experiment (*P* < 0.05) (Figure 7A). These results revealed that coumarin was able to treat *C. albicans* infection. To determine the toxicity of coumarin, non-infected mice were administrated with coumarin at doses of 20 and 40 mg/kg body weight by OG gavage. Data showed that all the mice survived the course of the experiment (Figure 7B), indicating that coumarin was not toxic below a dose of 40 mg/kg.

## Discussion

There is an urgent need to explore new-generation antifungal drugs due to the low efficacy, high toxicity, and drug resistance of the currently available antifungals (Carmona-Gutierrez et al., 2010). Many natural products such as lycopene, nerol, and limonene are reported to exhibit antifungal activity by inducing apoptosis. Apoptosis is a form of programmed cell death that is important for metazoan homeostasis and maintenance by eliminating unwanted, mutated, damaged, and superfluous cells (Rockenfeller and Madeo, 2008). In addition to metazoans, apoptosis has also been discovered in unicellular organisms such as *C. albicans*. Previous studies have demonstrated that coumarin and its derivatives display remarkable activities against fungi (Arshad et al., 2011). However, the potential mechanism explaining its antifungal action has not yet been fully elucidated.

In this study, we found that coumarin could inhibit cell growth and reduce strain viability. To study the pattern of cell death, the features of yeast apoptosis were determined, including PS externalization, DNA fragmentation, cytochrome *c* release, and metacaspase activation (Carmona-Gutierrez et al., 2010). The results showed that coumarin treatment led to PS exposure on the outer leaflet, DNA fragmentation and nuclear condensation, cytochrome *c* release from the mitochondria to the cytosol, and metacaspase activation, suggesting that coumarin induced apoptosis in *C. albicans*. Previous studies have demonstrated that coumarin damages *C. albicans* cells via pore formation in the cell wall and that cell death occurs due to the leakage of cytoplasmic contents and necrosis (Widodo et al., 2012), which is not consistent with our results. This might be due to the difference in the *C. albicans* strains used, distinct culture medium, incubation temperature, and coumarin treatment time.

As is known, mitochondria are both the origin and target of ROS (Terman et al., 2006), and ROS and mitochondria play an essential role in apoptotic processes (Pereira et al., 2008). During apoptosis, oxidative stress induces the mitochondrial permeability transition pore (PTP) to open, which then triggers the mitochondrial outer membrane permeabilization (MOMP) that is involved in the release of cytochrome *c* and other proapoptotic factors, leading to subsequent metacaspase activation (Simon et al., 2000). ROS can also directly induce apoptosis in a metacaspase-dependent manner (Wu et al., 2010). In this study, coumarin-treated *C. albicans* cells exhibited elevated ROS levels, decreased mitochondrial membrane potential, and abnormal mitochondrial morphology, indicating that coumarin triggered ROS accumulation and mitochondrial dysfunction.

Besides ROS, Ca^2+^ is also closely related to apoptosis (Carraro and Bernardi, 2016). In mammalian cells, apoptotic stimuli often cause an increase in cytosolic Ca^2+^ fueled via extracellular calcium influx and/or calcium release from intracellular stores (Jia et al., 2015). Among them, the release of Ca^2+^ from lysosomes is crucial for PS externalization (Mirnikjoo et al., 2009). Subsequently, cytosolic Ca^2+^ is rapidly relayed to mitochondria through MCU complex-dependent uptake (Almeida et al., 2008). Under oxidative stress, this Ca^2+^ signal can lead to PTP opening and release of cytochrome *c* and other proapoptotic factors, which ultimately results in the formation of apoptotic bodies and cell death. Although there is no MCU complex in yeast mitochondria, apoptotic stimuli could induce enough mitochondrial Ca^2+^ to undergo the permeability transition, which is accompanied by increased ROS and cytosolic Ca^2+^ levels. Once PTP opens, Ca^2+^ levels in the cytosol and matrix equilibrate and stabilize PTP in the open conformation, leading to matrix swelling, outer mitochondrial membrane damage, and release of proapoptotic proteins, thereby causing cell death (Carraro and Bernardi, 2016). Our study found that coumarin treatment caused Ca^2+^ accumulation in the cytosol and mitochondria, which might contribute to apoptosis.

Several studies reported that antioxidant or mitochondrial Ca^2+^ channel inhibitors could rescue cells from natural product-induced apoptosis (Zhang et al., 2008; Yun and Lee, 2016; Lee and Lee, 2017), indicating that oxidative stress or elevated mitochondrial Ca^2+^ might be the reasons leading to apoptosis. In our study, ROS removal did not prevent DNA fragmentation or metacaspase activation caused by coumarin, thereby demonstrating that coumarin-triggered apoptosis was independent of ROS or oxidative stress. However, the mitochondrial Ca^2+^ inhibitor significantly decreased coumarin-induced apoptosis-related features, suggesting that apoptosis was mediated by mitochondrial Ca^2+^ levels. Some coumarin derivatives have been reported to regulate mitochondrial permeability transition associated with cytosolic and mitochondrial Ca^2+^ equilibration (Cálgarohelena et al., 2006). When mitochondrial Ca^2+^ levels decreased, which was not enough to induce PTP opening, apoptosis was observed to be ameliorated. In summary, these results indicated that coumarin caused mitochondrial Ca^2+^-dependent apoptosis in *C. albicans*.

Singh et al. demonstrated that the coumarin derivative, SCD-1, could increase the survival of *Aspergillus fumigatus*-infected mice and decrease the colony counts in vital organs by treating with a dose of 200 mg/kg body weight orally or 100 mg/kg intraperitoneally, indicating that SCD-1 could be a potential strategy to treat aspergillosis (Singh et al., 2014). Previous studies mainly focused on the antifungal activity of coumarin or its derivatives against *C. albicans in vitro* (Thati et al., 2007; Creaven et al., 2009). The efficacy of these compounds *in vivo* is unclear. In this study, infected mice were administrated with coumarin by OG gavage to evaluate its anti-Candida activity *in vivo*. The results showed that the dose of 40 mg/kg coumarin prolonged the survival of the infected mice. Furthermore, toxicity assays showed that coumarin was non-toxic to the mice at the 40 mg/kg dose and low-toxic to human umbilical vein endothelial cells (HUVECs) (Figure S5). These results indicate that coumarin is effective and exhibits low toxicity in treating *C. albicans* infection.

## Ethics Statement

Healthy C57BL/6 mice (male: 18–22 g) were provided by Wenzhou Medical University (License No. SCXK [ZJ] 2005– 0019). The Guide for the Care and Use of Laboratory Animals of the China National Institutes of Health was followed for procedures involving animals. All procedures were approved by the Animal Care and Use Committee of Wenzhou Medical University (wydw 2017-0046). All efforts were made to minimize the suffering of the mice.

## Author Contributions

CJ and JZ were responsible for designing and working in most aspects of the work, including the analysis and interpretation of data and the drafting of the manuscript. LY, CW, and YY participated in measuring ROS, Ca^2+^ levels, cytochrome *c* contents, and metacasapse activity. XR carried out the experiments about Annexin-FITC/PI co-staining and TUNEL staining. KX observed mitochondrial morphology and DNA fragmentation using Mito-Tracker and DAPI staining. MC participated in revising the manuscript and supervised the work.

### Conflict of Interest Statement

The authors declare that the research was conducted in the absence of any commercial or financial relationships that could be construed as a potential conflict of interest.

## Supplementary Material

Figure S1 The MDA contents were measured in *C. albicans* after treatment with coumarin for 4 h.

Figure S2 The MIC values of coumarin for 04311 and ATCC18804 strains were measured. Data were shown as mean ± SD. ^*^*P* < 0.05 and ^**^*P* < 0.01.

Figure S3 Coumarin triggered metacaspase activation related to mitochondrial Ca^2+^ influx in 04311 and ATCC18804 strains. The histogram displayed the percentage of stained cells, and the data were exhibited as mean ± SD, ^*^*P* < 0.05, ^**^*P* < 0.01 and ^***^*P* < 0.001.

Figure S4 Coumarin induced DNA fragmentation associated with mitochondrial Ca^2+^ influx in 04311 and ATCC18804 strains. The percentage of stained cells was shown in the histogram, and the data were displayed as mean ± SD. ^**^*P* < 0.01 and ^***^*P* < 0.001.

Figure S5 Effect of coumarin on the survival of human umbilical vein endothelial cells (HUVECs). Data were shown as mean ± SD, ^*^*P* < 0.05.

## Abbreviations

ROS: reactive oxygen species
DCFH-DA, 2′: 7′-dichlorodihydro-fluorescein diacetate
PI: propidium iodide
DAPI: 4′-6-diamino-2-phenylindole
CFU: colony forming units
MIC: minimal inhibitory concentration
JC-1, 5, 5′, 6, 6′-Tetrachloro-1, 1′, 3: 3′-tetraethyl-benzimidazolyl carbocyanine iodide.
