# Supplementary figures and images for: Antifungal Activity of Coumarin Against Candida albicans Is Related to Apoptosis
Source: Front Cell Infect Microbiol. 2019 Jan 4;8:445. doi: 10.3389/fcimb.2018.00445 (PMC6328497; doi:10.3389/fcimb.2018.00445)

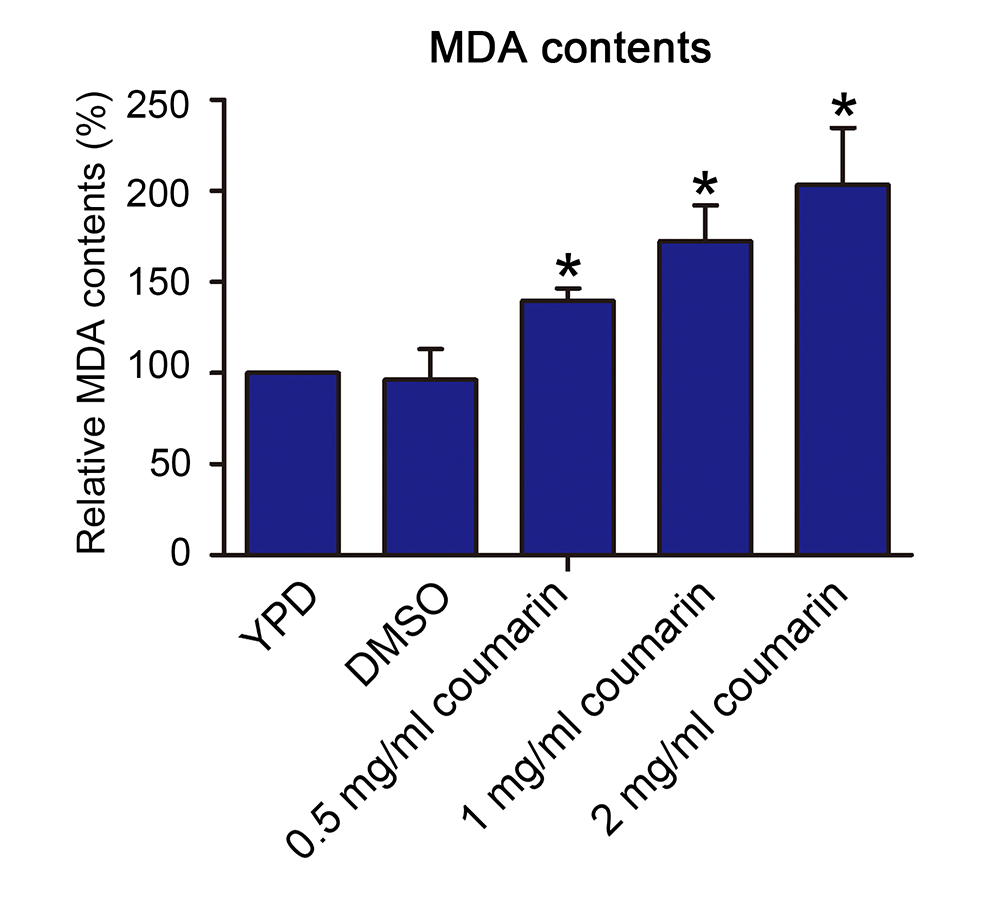

Supplement: Figure S1 — The MDA contents were measured in C. albicans after treatment with coumarin for 4 h. [file Image_1.JPEG]

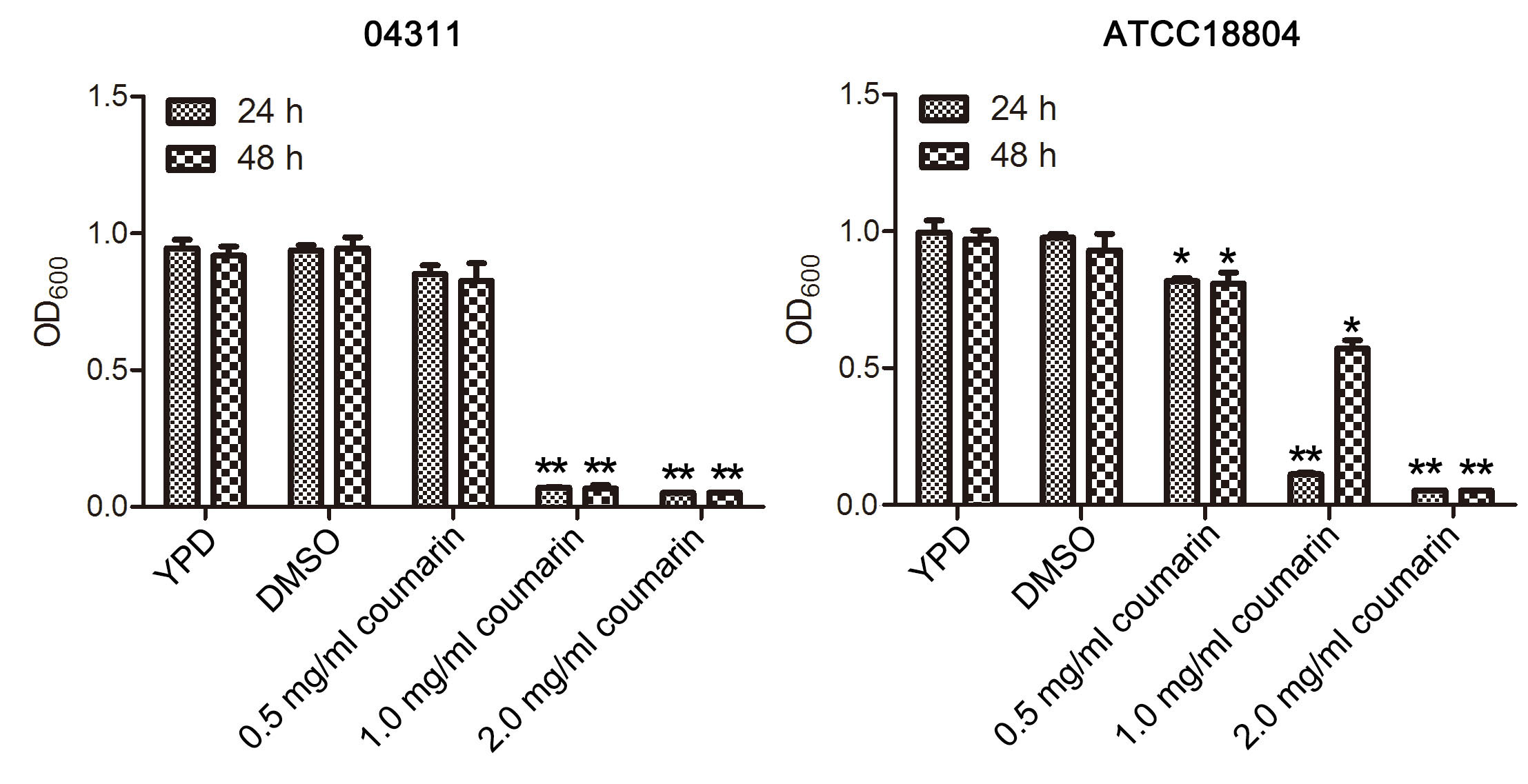

Supplement: Figure S2 — The MIC values of coumarin for 04311 and ATCC18804 strains were measured. Data were shown as mean ± SD. *P < 0.05 and **P < 0.01. [file Image_2.JPEG]

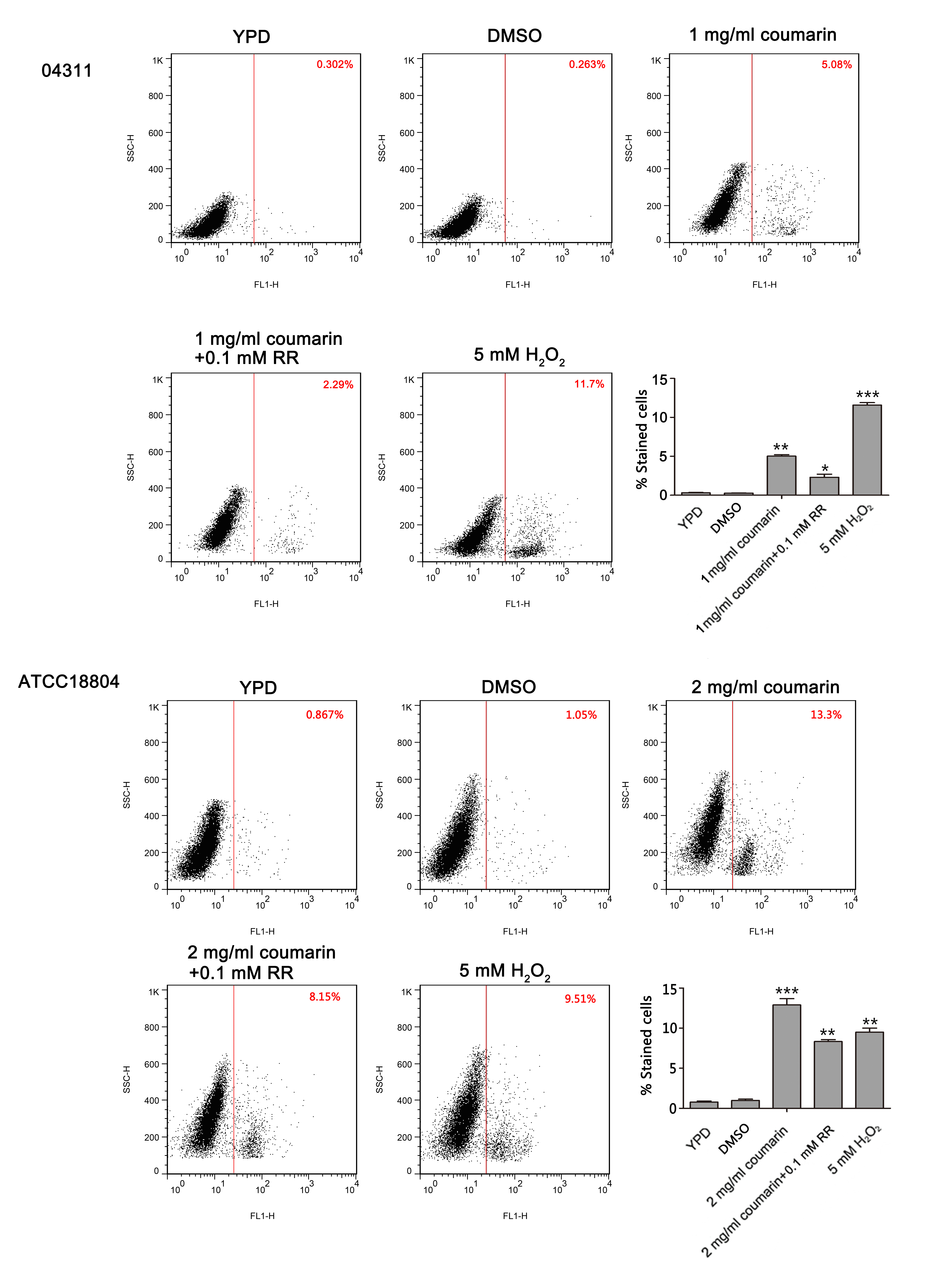

Supplement: Figure S3 — Coumarin triggered metacaspase activation related to mitochondrial Ca2+ influx in 04311 and ATCC18804 strains. The histogram displayed the percentage of stained cells, and the data were exhibited as mean ± SD, *P < 0.05, **P < 0.01 and ***P < 0.001. [file Image_3.JPEG]

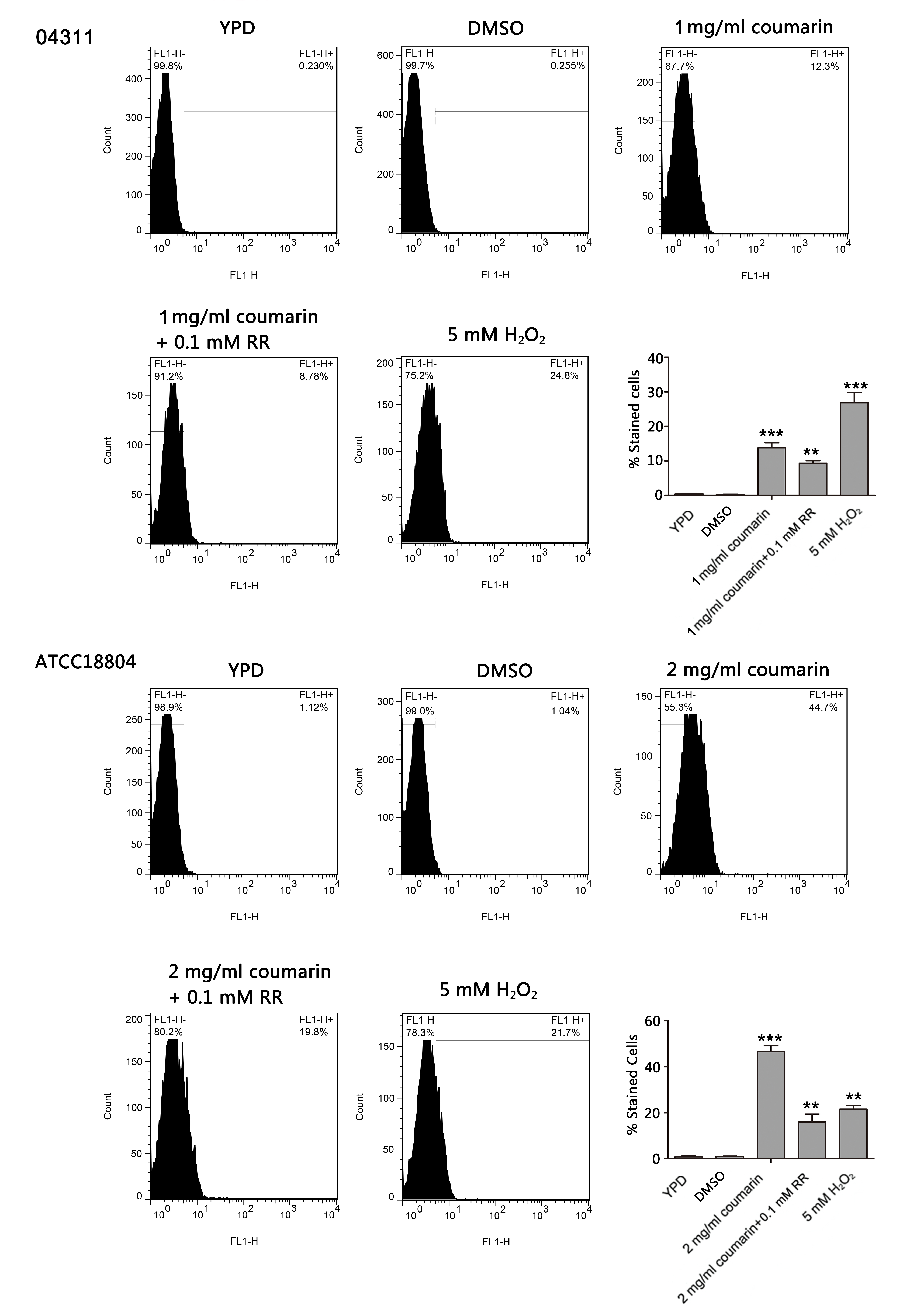

Supplement: Figure S4 — Coumarin induced DNA fragmentation associated with mitochondrial Ca2+ influx in 04311 and ATCC18804 strains. The percentage of stained cells was shown in the histogram, and the data were displayed as mean ± SD. **P < 0.01 and ***P < 0.001. [file Image_4.JPEG]

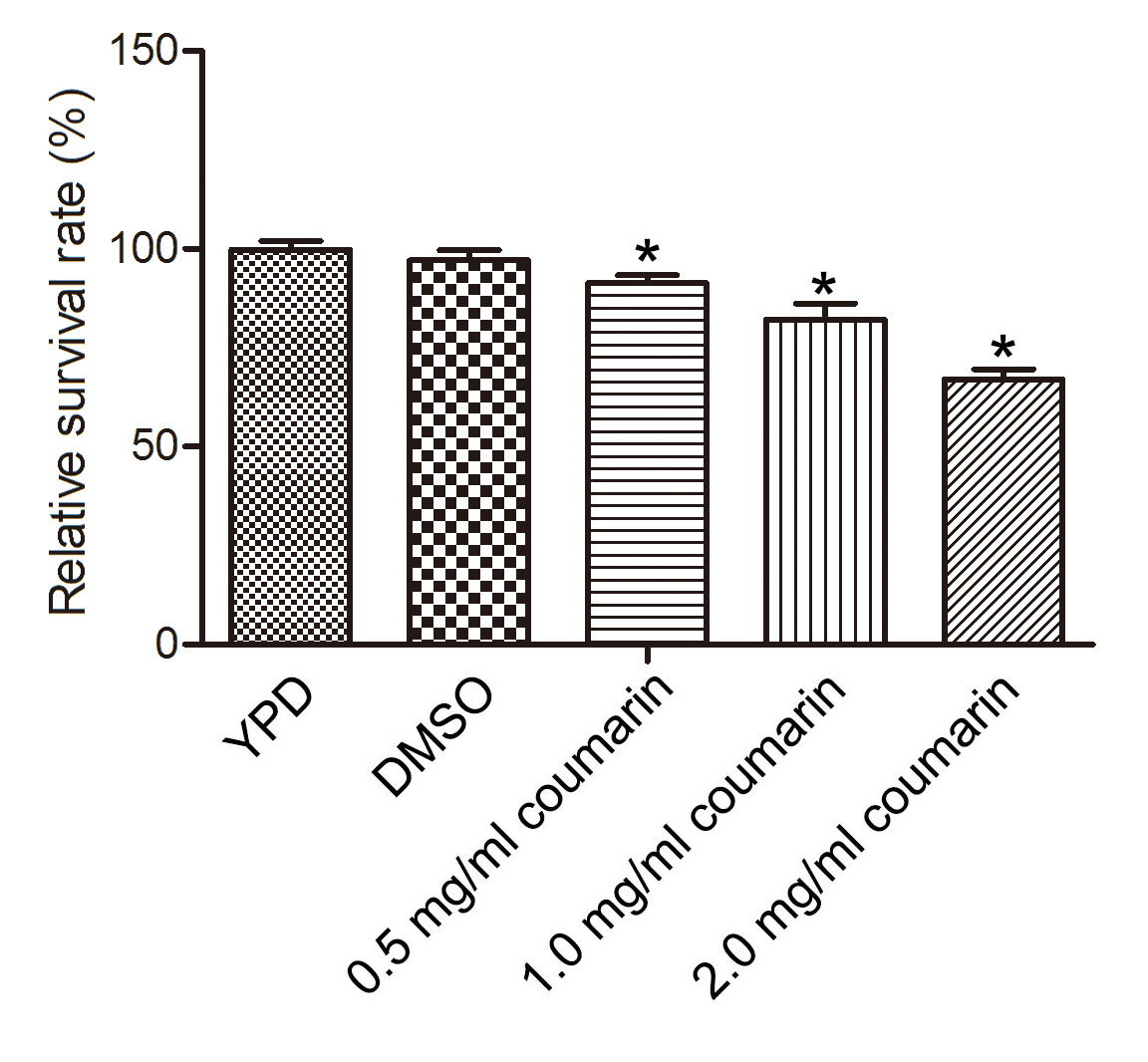

Supplement: Figure S5 — Effect of coumarin on the survival of human umbilical vein endothelial cells (HUVECs). Data were shown as mean ± SD, *P < 0.05. [file Image_5.JPEG]
